# Extracellular neutrophil traps: a novel therapeutic target in ANCA-associated vasculitis?

**DOI:** 10.3389/fimmu.2013.00024

**Published:** 2013-02-04

**Authors:** Andreas P. Diamantopoulos

**Affiliations:** ^1^Department of Rheumatology, Hospital of Southern Norway Trust KristiansandKristiansand, Norway; ^2^Department of Neurosciences, Faculty of Medicine, Norwegian University of Science and TechnologyTrondheim, Norway

**A commentary on**

**Abundant neutrophil extracellular traps in thrombus of patient with microscopic polyangiitis**

by Nakazawa et al. (2012). Front. Immun. 3:333. doi: 10.3389/fimmu.2012.00333

Extracellular neutrophil traps (NETs) have gathered lots of attention in recent years with regard to pathogenesis of diverse inflammatory and autoimmune diseases [gout, systemic lupus erythematosus (SLE), and vasculitis] (Kessenbrock et al., 2009; Bosch, 2011; Mitroulis et al., 2011). It seems that neutrophils are not only basic players and mediators of innate immunity, but are also involved in activation, regulation, and effector functions of adaptive immune cells (Mantovani et al., 2011).

Neutrophils are considered as the mainstay of cellular innate immune responses. During the infection, numerous neutrophils infiltrate the affected tissue, trying to eradicate the bacteria by phagocytosis (Nathan, 2006), and generate chemotactic signals that attract monocytes and dendritic cells (Bennouna et al., 2003). However, occasionally, the above mechanism seems to be ineffective *in vivo* due to the flow dynamics of circulation (Phillipson and Kubes, 2011). Thus, neutrophils have adopted other more effective defense mechanisms. They can release their DNA in the presence of bacteria-creating traps called NETs—a weave of DNA fibers consisting of histones and antimicrobial proteins [myeloperoxidase (MPO), elastase, proteinase 3 (PR3)]—with a strong antibacterial effect (Brinkmann et al., 2004). Nevertheless, besides the beneficial role during infections, NETs can injure the endothelium leading to irreversible damage (Xu et al., 2009). In addition, NETs can serve as autoantigens that activate the plasmacytoid dendritic cells (pDCs) that in turn trigger B cells' activation, enhancing by this way autoantibody production (Lande et al., 2011).

In the present case study (Nakazawa et al., 2012b), the abundant existence of NETs in a thrombus of a patient who died from a fulminant multi-organ microscopic polyangiitis (MPA) is described for the first time. In addition, the study confirms previous reports showing presence of NETs in the glomeruli in kidneys affected by MPA (Kessenbrock et al., 2009). It appears that the presence of thrombocytes is essential for the formation of NETs. Thrombocytes can bind to different types of leucocytes enhancing the production of numerous inflammatory molecules (Semple et al., 2011). Activated neutrophils alone do not appear to damage the endothelium, however, the binding of stimulated thrombocytes to neutrophils induces endothelial cell-death through the development of NETs (Clark et al., 2007). In turn, NETs provide a framework for thrombocytes' adhesion, leading to firm thrombus formation (Fuchs et al., 2010; Nakazawa et al., 2012b).

In the renal tissue of patients suffering from systemic small-vessel vasculitis, NETs have been shown to be directly involved in the endothelial damage caused by anti-neutrophil cytoplasmic antibodies (ANCA) (Kessenbrock et al., 2009). NETs seem to substantially enhance the uptake of PR3 and MPO molecules by myeloid dendritic cells (mDCs), leading to a significant induction of ANCA production and subsequent endothelial damage in renal and pulmonary tissue in mice (Sangaletti et al., 2012). In another study by Nakazawa et al. the use of propylthiouracil in rat neutrophils caused induction of abnormal NETs and led to the further development of MPO-ANCA and pulmonary capillaritis (Nakazawa et al., 2012a). Sangaletti et al. showed that inhibition of the NETs' production with deoxyribonuclease (DNAse), which selectively digests NETs DNA, prevented loading of PR3 and MPO by mDCs and led to inhibition of vasculitis.

The existence of NETs in the different organs affected by vasculitis raises an interesting question of whether or not the neutrophils undergoing cell-death by NETs could be a worthwhile therapeutic target. Taking into account all of the above, it seems that in pathological neutrophil activation, targeting the extracellular DNA and histones in NETs by DNAse may be beneficial by preventing thrombosis and irreversible end-organ damage induced by vasculitis. In addition, inhibition of peptidelargine deaminase 4 (PAD4), which citrullinates histones, and nicotinamide adenine dinucleotide phosphate (NADPH) oxidase-which generates reactive oxygen species—both essential for NETs formation, could be an interesting therapeutic target (Nakazawa et al., 2012b).

The study of Nakazawa et al. is also important for another reason. It reminds us of how difficult can be the diagnosis of the ANCA-associated vasculitis presenting with dramatic manifestations of multi-organ failure. In these patients, immediate initiation of treatment is crucial in order to diminish the end-organ damage and protect the vital functions of the affected individual. However, the meticulous clinician should always keep in mind that intense signs of inflammation and multi-organ failure can also be seen in other conditions, such as infections.

In conclusion, further studies are needed to explore the therapeutic implication of targeting NETs in the ANCA-associated vasculitis. Prompt initiation of treatment in patients suffering from ANCA-associated vasculitis can secure the best outcomes.
